# Human placenta as a biological vascular model for endovascular training

**DOI:** 10.3389/fneur.2026.1856017

**Published:** 2026-09-03

**Authors:** Z. Itsekzon-Hayosh, E. Chung, B. Warren, N. Agid, A. Helmi, S. C. Mafeld, P. R. Mosimann

**Affiliations:** 1 Joint Department of Medical Imaging, University Health Network, Toronto, ON, Canada; 2 Department of Medical Imaging, Divisions of Neuroradiology and Vascular and Interventional Radiology, University of Toronto, Toronto, ON, Canada

**Keywords:** embolization, endovascular treatment, neurointervention, simulation, stenting, thrombectomy, training

## Abstract

**Introduction:**

Simulation-based training in endovascular procedures remains limited by the availability of biological vascular models that provide tactile feedback. Existing animal, cadaveric, synthetic, and virtual simulators incompletely reproduce the mechanical behavior of complex neurovascular anatomy.

**Methods:**

Human placentas were prepared by cannulation of the umbilical vessels, vascular flushing, and establishment of continuous gravity-driven flow. Vessel curvature was modified to simulate challenging neurovascular anatomy, including carotid siphon—like configurations, and aneurysm analogues were created. Controlled vessel perforations were performed to simulate emergent hemostatic scenarios. Endovascular procedures included liquid embolization with ethylene—vinyl alcohol copolymer (EVOH) and n-butyl cyanoacrylate (NBCA), coil embolization, flow-diverting stent deployment, and endovascular retrieval techniques. All procedures were performed under angiographic guidance with continuous flow.

**Results:**

The placenta model provided reliable angiographic visualization and tactile feedback. Liquid embolization demonstrated procedural behaviors including reflux, plug formation, distal penetration, and effective hemostasis. Stent deployment produced vessel straightening with subsequent elastic recoil. Thrombus and device retrieval simulations demonstrated vessel deformation with recovery after device removal.

**Conclusion:**

The human placenta represents an ethically favorable biological platform for endovascular training. This model enables simulation of a broad range of neurointerventional and vascular procedures while reducing reliance on animal models.

## Introduction

Endovascular therapies encompass a broad range of techniques, including intravascular stent placement, embolization using coils, plugs, and liquid embolic agents, and mechanical thrombectomy. Mastery of these procedures requires both detailed anatomical knowledge and familiarity with device behavior, deployment mechanics, and bailout strategies. Most procedural experience is currently acquired under direct supervision in the clinical setting, with limited opportunities for structured, deliberate practice in a controlled environment.

Simulation-based approaches for endovascular training have traditionally relied on animal models, human cadavers, and synthetic large-vessel simulators (1–3). Virtual reality—based platforms have more recently expanded training opportunities, primarily for cardiac, thoracoabdominal, peripheral, and basic neurovascular interventions. While these systems offer high-quality visual representation, they provide limited tactile feedback and do not replicate vessel compliance, both of which are critical to endovascular skill acquisition (3).

To address these limitations, the human placenta has been explored as a biological vascular model for endovascular and microvascular simulation. Early studies demonstrated the feasibility of the placenta as an *ex vivo* neurointerventional platform based on angiographic visibility and mechanical similarity to cerebral vessels (4). Subsequent work established face, content, and construct validity, demonstrating realistic visual and haptic feedback across a range of neurovascular tasks (5, 6). More recently, the placenta has been adapted for mechanical thrombectomy training, further expanding its applicability (7).

Placental models have also been adapted for other vascular and microsurgical tasks. These include pulsatile-flow systems for aneurysm clipping and vascular anastomosis, intraluminal thrombi for microsurgical thrombectomy, and systematic mapping of placental vessels for procedure-specific training (8–11). More recent models have combined a perfused placenta with a cadaveric head for aneurysm surgery (12) and recreated arteriovenous malformations with continuous or pulsatile flow (13).

Despite these advances, key aspects of neurointerventional practice, including flow optimization, vessel manipulation, liquid embolic behavior, and bailout retrieval techniques, require further evaluation to support structured and reproducible training.

In this feasibility study, we evaluated the human placenta as a biological vascular model for endovascular and microvascular training without animal sacrifice. The long-term objective is integration of this model into structured training curricula for interventional neuroradiology and vascular and interventional radiology trainees. We specifically assessed intravascular flow optimization, vessel modification to simulate neurovascular anatomy, simulation of liquid embolic techniques including perforation management, and endovascular retrieval procedures.

## Materials and methods

### Placenta preparation

Fresh placenta was collected immediately after cesarean delivery, with the umbilical cord and its vessels clamped according to the standard operating procedure. The placenta was weighed and assessed for integrity. The placenta was then gently washed with non-sterile water to remove surface-adherent blood. Subsequently, the placenta was placed on an absorbent pad, and the amnion was dissected from the chorionic plate, revealing the superficial vessels of the chorionic plate (Supplementary Figure S1A). The umbilical cord was shortened to 5–10 cm and blood was drained and manually expressed from the chorionic vessels. A total of 11 placental specimens were obtained from the institutional biobank; three were used for protocol development and excluded, leaving eight placentas included in the study.

All three umbilical vessels were cannulated directly at their cross-sectional orifices using a 4-French dilator (Supplementary Figure S1B). Each umbilical vessel was manually flushed until complete clearance of blood was achieved, followed by infusion of heparinized saline (1,000 U/L). A 0.035-inch short guidewire was advanced through the dilator, then exchanged for 16-gauge angiocatheter. This process was repeated for all three vessels, and the angiocatheters were secured using an umbilical clamp to maintain access (Supplementary Figure S1C). Prepared placentas were stored at 5 °C for up to two nights prior to simulation, with angiocatheters left *in situ* to preserve vascular access. This protocol was refined during the initial three placentas used for protocol development.

### Placenta preparation for angiographic assessment and flow setup

Prior to simulation, the placenta was transferred to a custom-made tray designed with a gentle downward slope toward an outflow port connected to a drainage bag, allowing continuous gravity-driven outflow (Supplementary Figure S2A). The umbilical clamp was removed, and each 16-gauge angiocatheter was exchanged over the mini-guidewire for a 6- or 8-French vascular sheath (Supplementary Figure S2B). The sheath—vessel interfaces were sealed using cyanoacrylate glue to prevent leakage. Continuous saline flow was established through the sheaths via a non-pressurized saline bag, or through a guiding catheter when present, to allow ongoing washout during angiographic acquisition (Supplementary Figure S2C).

### Vessel curvature and aneurysm creation

Selected placental vessels were modified to simulate challenging neurovascular anatomy by placing a 6–0 polypropylene suture through placental tissue adjacent to the selected vessel segment and applying traction to create an exaggerated angulation resembling a carotid siphon configuration (Supplementary Figure S3A). Sidewall aneurysm analogues were created by ligating a branch vessel using absorbable suture, forming an aneurysmal stump along the parent vessel (Supplementary Figure S3B).

### Perforation creation

Controlled vessel perforations were created to simulate acute vascular injury (Supplementary Figure S4). Endoluminal perforations were produced by advancing the stiff end of a microwire (0.014–0.021 inch) through the vessel wall, while alternative perforations were created using an external approach with a 22-gauge angiocatheter needle.

### Image acquisition

All procedures were performed in a clinical angiography suite using a Philips Azurion 7 M20 monoplane angiography system (Philips Healthcare, Amsterdam, Netherlands). Standard digital subtraction angiography, roadmap guidance, and blank roadmapping were used. Iohexol contrast agent (Omnipaque 300, GE Healthcare, Mississauga, Canada) was used for angiographic imaging. Contrast clearance was achieved through continuous saline washout driven by gravitational drainage through the placental tissue and confirmed fluoroscopically.

### Liquid embolics

Liquid embolic materials were introduced via Sonic (Balt Ltd., Montmorency, France) and Progreat (Terumo, Tokyo, Japan) microcatheters. We used either ethylene vinyl alcohol copolymer (EVOH), dimethyl-sulfoxide (DMSO) and micronized tantalum powder (TA)-based products (Onyx by Medtronic, Minneapolis, MN, USA or Squid by Balt Ltd., Montmorency, France), or n-butyl 2-cyanoacrylate (NBCA) + metacryloxysulfolane (MS) based products (Glubran 2, GEM Ltd., Viareggio, Italy). The NBCA-based material was mixed with Lipiodol at concentrations of 33, 50, and 66% prior to injection. Microcatheters used for EVOH injections were primed with dimethyl sulfoxide, whereas those used for NBCA injections were flushed with 5% dextrose solution. Guiding catheters used for NBCA delivery were connected to a continuous saline flush containing 10% bovine serum albumin solution (weight/volume) to facilitate polymerization (14). All experiments were conducted at room temperature (22–25 °C).

### Coiling and flow diversion

Standard clinical microcatheters and microwires were used for coil embolization and deployment of flow-diverting and conventional intracranial stents. Retrieval simulations included deliberate stent malpositioning followed by retrieval using stent retriever devices. Mock thrombi composed of placental blood clots and synthetic material were used for thrombectomy simulations.

### Ethics statement

All experiments were conducted in accordance with institutional research ethics requirements. Placental specimens were obtained through an institutional biobank with active research ethics board approval (#: 10-0128-E). Written informed consent was obtained from all donors, and all specimens were de-identified prior to use.

## Results

Contrast injection into the umbilical vessels provided clear angiographic visualization of the placental vascular network and enabled roadmap-guided navigation through the chorionic plate vessels (Supplementary Figure S5). Continuous saline washout allowed repeated angiographic acquisitions without significant contrast retention.

**Figure 1:**
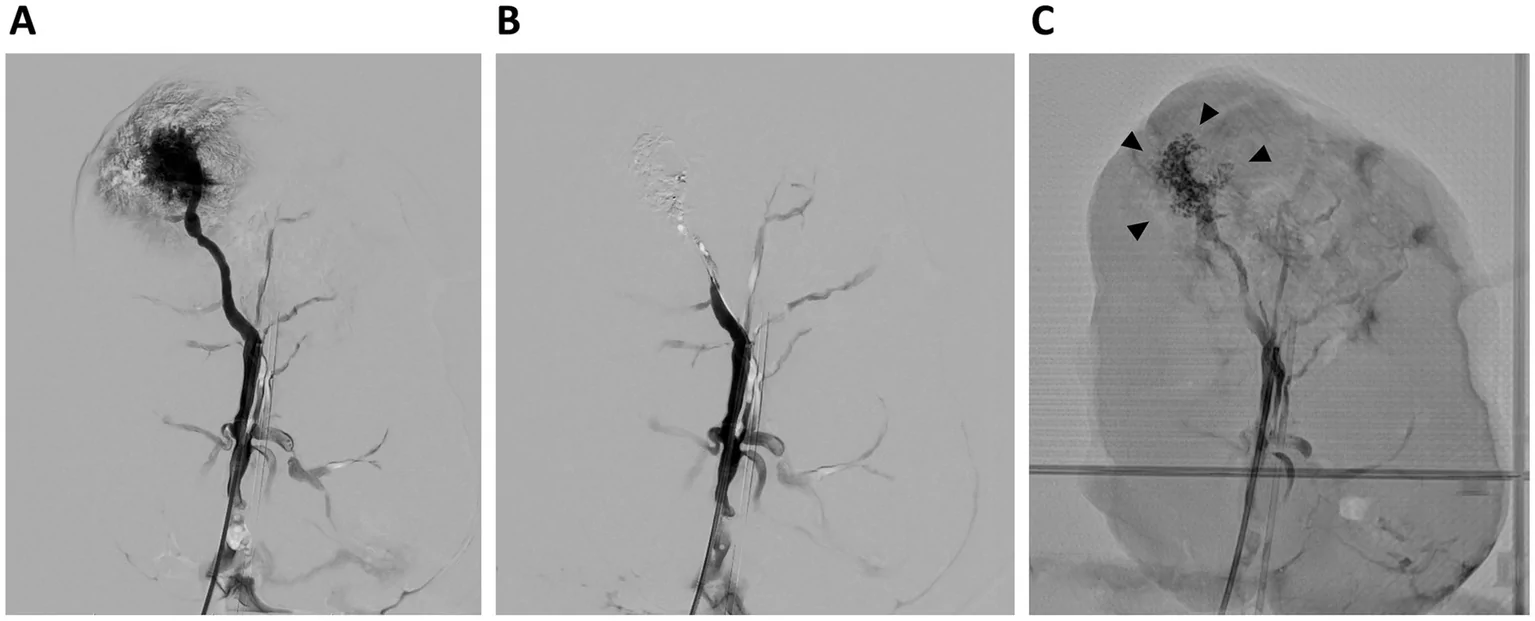
Vessel perforation and glue embolization in the placental vascular model. **(A)** Selective angiogram of perforated vessel. **(B)** Subtracted angiogram of selected vessel post glue embolization. **(C)** Fluoroscopy of the placenta demonstrating the glue cast (arrowheads).

Controlled vessel perforation resulted in focal contrast extravasation, simulating acute vessel injury (Figure 1A). Hemostasis was successfully achieved using NBCA embolization, with progressive occlusion of the perforation site and cessation of extravasation on subsequent angiographic runs (Figures 1B,C).

**Figure 2:**
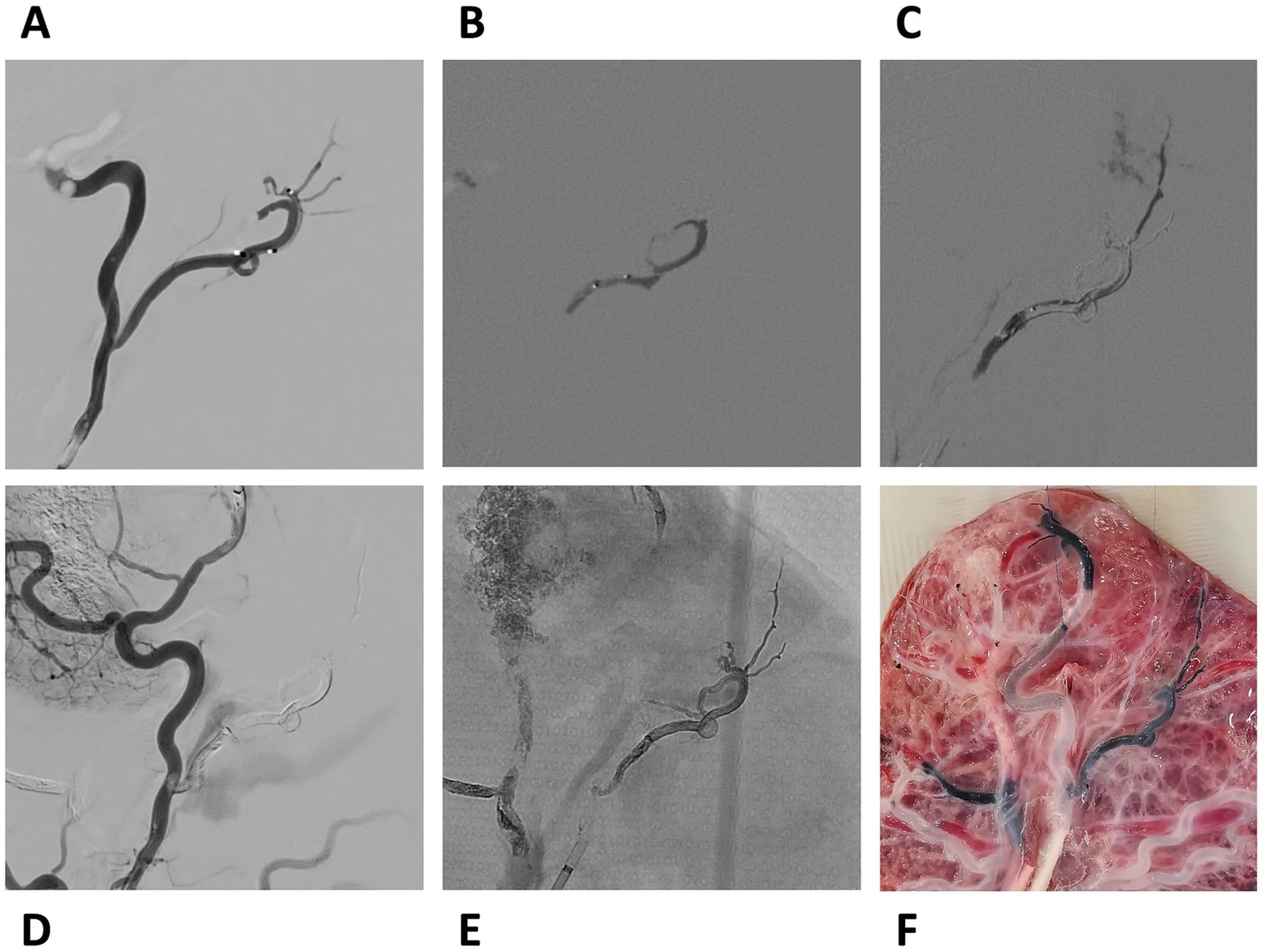
EVOH liquid embolization in the placental vascular model. **(A)** Microcatheter angiogram confirming catheter position. **(B)** Blank roadmap depicting EVOH injection with parent feeder reflux. **(C)** Distal penetration into the chorionic capillary bed. **(D)** Subtracted angiogram after EVOH embolization. **(E)** Fluoroscopy of the placenta demonstrating the EVOH cast. **(F)** Direct visualization of the embolized chorionic plate vessel.

For EVOH embolization, a DMSO-compatible microcatheter (Sonic 1.2F, Balt Ltd., Montmorency, France) was navigated distally into the chorionic plate artery, followed by a microinjection to confirm catheter position (Figure 2A). The microcatheter was flushed with DMSO, and Onyx 18 (Medtronic, Minneapolis, MN, USA) was then injected. Initial reflux into the parent feeder vessel was observed (Figure 2B), followed by plug formation and distal penetration into the chorionic capillary bed (Figure 2C). Angiography demonstrated embolization of the chorionic plate vessel (Figures 2D,E). Direct visualization of the embolized vessels is shown in Figure 2F.

**Figure 3:**
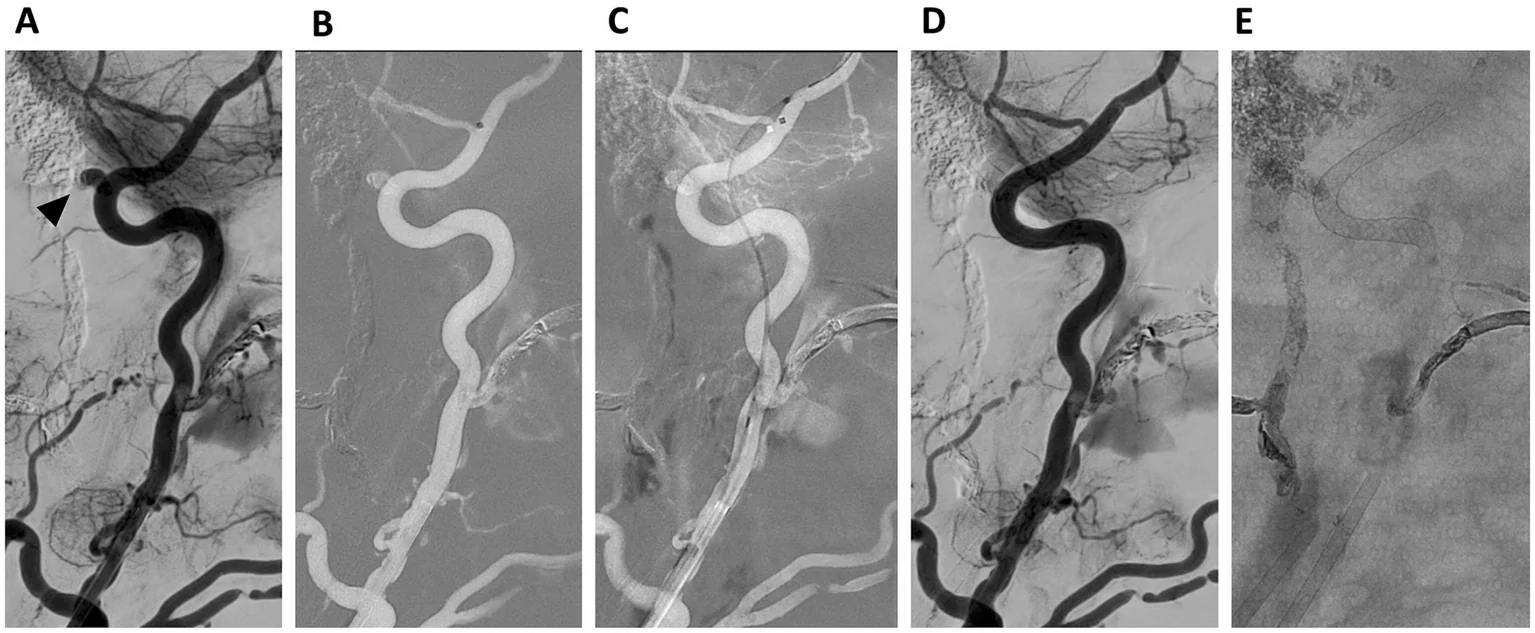
Flow diverter deployment in the placental vascular model. **(A)** Angiogram demonstrating exaggerated vessel curvature and a sidewall aneurysm (arrowhead). **(B)** Phenom 27 microcatheter navigated distal to the aneurysm. **(C)** Constrained flow diverter within the microcatheter. **(D)** Angiogram demonstrating non-filling of the aneurysm after flow diverter deployment. **(E)** Deployed flow-diverting stent.

Following aneurysm creation with exaggerated vessel curvature (Figure 3A), a 0.027″ microcatheter (Phenom 27; Medtronic, Minneapolis, MN, USA) was navigated distal to the aneurysm (Figure 3B). A 4.5 mm × 20 mm pipeline flow-diverting stent (Medtronic, Minneapolis, MN, USA) was deployed across the aneurysm into the parent vessel, resulting in non-filling of the aneurysm on the control angiographic run (Figure 3). During delivery, the parent vessel demonstrated straightening with subsequent elastic recoil following stent deployment.

**Figure 4:**
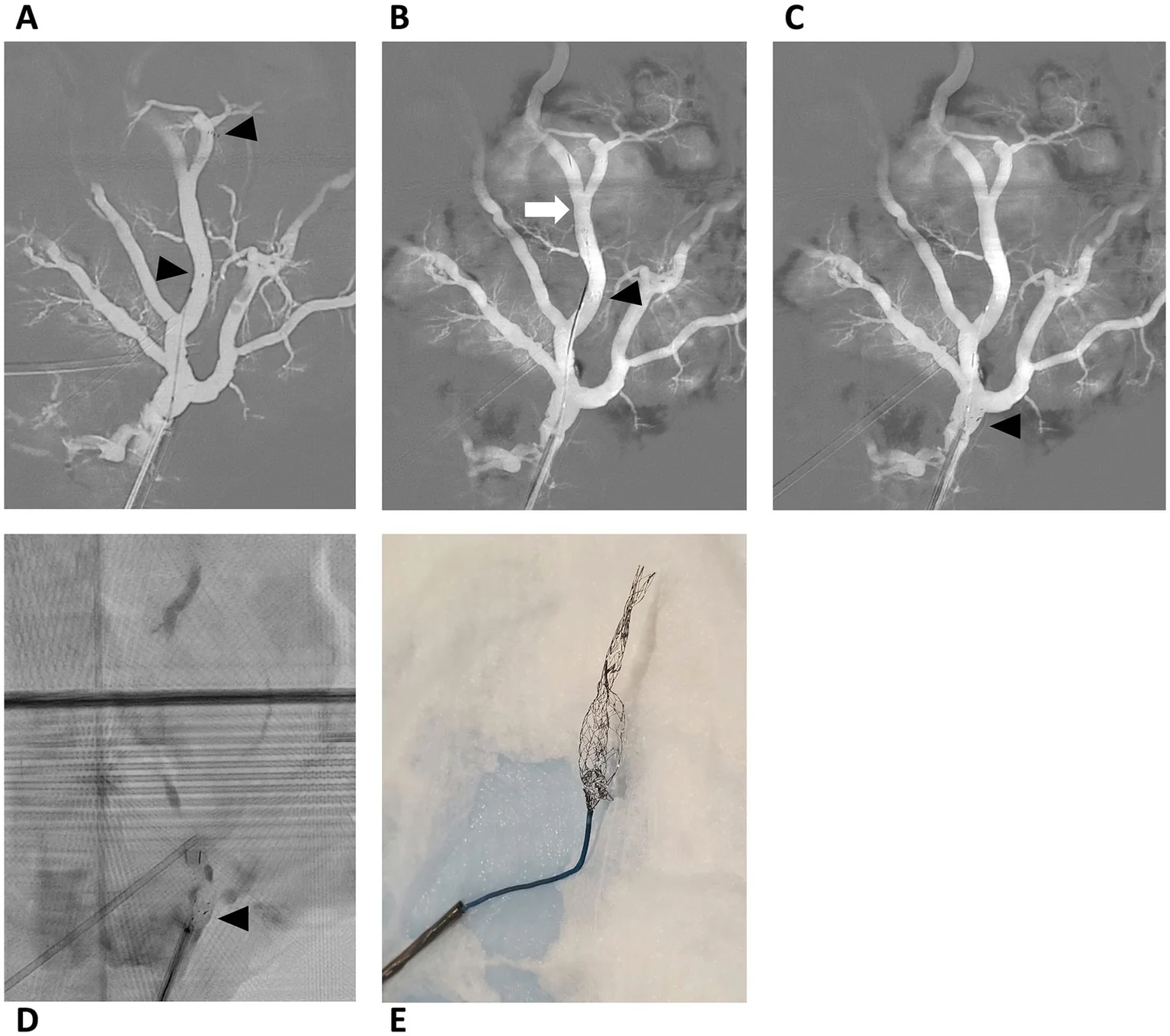
Retrieval of a malpositioned stent in the placental vascular model. **(A)** Deliberately malpositioned Enterprise stent with distal and proximal markers visualized on roadmap imaging (arrowheads). **(B)** Positioning of the EMBOTRAP III device (white arrow) distal to the Enterprise stent (arrowhead) prior to retrieval. **(C)** Enterprise stent (arrowhead) captured within the guide sheath with the EMBOTRAP III device. **(D)** Fluoroscopy demonstrating the captured Enterprise stent (arrowhead) within the guide sheath without roadmap overlay. **(E)** Retrieved Enterprise stent captured by the EMBOTRAP III device.

Retrieval simulations were performed for both malpositioned stents and simulated thrombus. Deliberate stent malpositioning was followed by deployment of a stent retriever distal to the stent, enmeshment, and successful retrieval (Figures 4A–D). Thrombectomy simulations using mock synthetic clots (FLARP) resulted in successful retrieval in all attempts using standard stent retrievers, with visible vessel deformation during traction and recovery after device removal.

## Discussion

This feasibility study demonstrates that the human placenta can serve as a practical and versatile biological model for endovascular training, particularly for simulation of liquid embolization, aneurysm treatment, and retrieval techniques. The model demonstrated key procedural behaviors relevant to EVOH- and NBCA-based embolization, including polymerization dynamics under albumin-mediated conditions (14, 15). These applications complement prior placenta-based models used for microsurgical aneurysm treatment, vascular anastomosis, thrombectomy, and other vascular training tasks (8–13).

To our knowledge, this represents the first demonstration of deployment of various endovascular stents in an *ex vivo* placental vascular model. One notable feature of the placental vessels was vessel straightening during advancement of the stent delivery catheter, followed by elastic relaxation of vessel curvature after stent deployment. Another notable feature of the model is the ability to increase vessel curvature using simple suture traction, allowing simulation of challenging vascular anatomy for flow diverters, open- and closed-cell stents, and stent retrievers. While various *in vitro* models (made of silicone, resins, etc.) can reproduce vascular geometry, they often fail to replicate the flexibility, fragility, and mechanical responsiveness of living vessels. In contrast, the placental vessels demonstrated flexibility, fragility, and responsiveness during device manipulation. The creation of sidewall aneurysm analogues further enabled simulation of aneurysm treatment with flow diversion and coil embolization.

Simulation of retrieval techniques is particularly relevant given the clinical importance of bailout maneuvers in cases of device malposition or failure. In the present study, these techniques were simulated through retrieval of deliberately malpositioned intracranial stents and mock thrombi. Clinical series describing intracranial stentectomy and displaced device retrieval highlight the technical complexity and potential risks of these procedures (16, 17). Simulation platforms that allow operators to practice such techniques in a controlled setting may therefore represent an important adjunct to neurointerventional training.

## Limitations

### Reproducibility and standardization

Placental vascular anatomy varies between specimens. Standardization of specimen preparation, cannulation, sheath fixation, and open-loop drainage enabled reliable angiographic visualization and procedural execution. Formal assessment of reproducibility was beyond the scope of this feasibility study. Assessment of tactile feedback and procedural realism was based on the qualitative impressions of experienced operators and was not formally validated.

### Technical limitations

The main challenge in such a model, especially for liquid embolic simulation, is the reproduction of physiologic flow. In the present model, flow was generated through gravity-driven drainage and continuous saline infusion through the sheath or guiding catheter. Positioning the placenta on a tilted support allowed fluid to traverse the vascular network and exit through an open drainage pathway. This open-loop system provided sufficient flow for angiographic visualization and simulation of liquid embolic procedures, but did not reproduce physiologic pulsatility. Additional limitations of the *ex vivo* model include the absence of active vascular tone, endothelial metabolism, and physiological responses to vessel injury, as well as the use of room-temperature conditions for all experiments.

### Practical and logistical limitations

Logistical limitations include specimen availability, limited storage time, and challenges associated with handling human biological tissue. These factors may restrict implementation at institutions without access to placental specimens or biobanking support.

Nevertheless, clinically relevant phenomena such as reflux, plug formation, distal penetration, and polymerization behavior were observed despite non-physiologic, non-pulsatile flow, suggesting that pulsatility is not essential to reproduce these behaviors in this model.

Despite these limitations, the human placenta provides a practical and ethically acceptable platform for a wide range of endovascular simulation scenarios. Further work should focus on flow optimization, temperature control, and procedural standardization to support broader integration into endovascular training programs.

## Conclusion

The present study demonstrates the feasibility of using the human placenta as a single *ex vivo* platform for a range of endovascular training tasks, including liquid embolization, flow diversion, thrombectomy, perforation management, and device retrieval. The model may serve as a practical and ethically favorable complement to existing simulation systems.

## Data Availability

The original contributions presented in the study are included in the article/Supplementary material, further inquiries can be directed to the corresponding author.
