# Supplementary material for: Human placenta as a biological vascular model for endovascular training
Source: Front Neurol. 2026 Sep 3;17:1856017. doi: 10.3389/fneur.2026.1856017 (PMC13581842; doi:10.3389/fneur.2026.1856017)
Supplement: Supplementary file 1 [file Data_Sheet_1.DOCX]

**Supplementary Figures**

**Supplementary Figure 1.** Placenta preparation and vessel cannulation. (A) Removal of the amnion from the chorionic plate. (B) Cannulation of the umbilical vessels for flushing. (C) Prepared placenta with an umbilical clamp secured over the umbilical cord with guidewires and angiocatheters in place.

**Supplementary Figure 2.** Placenta setup for angiographic simulation. (A) Tray positioned with a downward slope and connected urinary drainage bag for continuous outflow. (B) Vascular sheaths secured within the umbilical vessels using cyanoacrylate glue. (C) Normal saline flush connected to the guiding catheter.

**Supplementary Figure 3.** Placental vessel modification for neurovascular simulation. (A) Suture placement used to create a siphon-like vessel curvature. (B) Suture placement used to create a sidewall aneurysm (arrowhead).

**Supplementary Figure 4.** Distal placental vessel perforation created with the stiff end of a microwire.

**Supplementary Figure 5.** Angiogram of the placental vessels.
